# ‘Fit Moms/*Mamás Activas*’ internet-based weight control program with group support to reduce postpartum weight retention in low-income women: study protocol for a randomized controlled trial

**DOI:** 10.1186/s13063-015-0573-9

**Published:** 2015-02-25

**Authors:** Suzanne Phelan, Anna Brannen, Karen Erickson, Molly Diamond, Andrew Schaffner, Karen Muñoz-Christian, Ana Stewart, Teresa Sanchez, Vanessa C Rodriguez, Dalila I Ramos, Linda McClure, Caro Stinson, Deborah F Tate

**Affiliations:** 1 grid.253547.2000000012222461XKinesiology Department, California Polytechnic State University, 1 Grand Avenue, San Luis Obispo, CA 93407 USA; 2 grid.10698.360000000122483208Department of Health Behavior, University of North Carolina Gillings School of Global Public Health, 318 Rosenau Hall, Campus Box 7400, Chapel Hill, NC 27599-7440 USA; 3 grid.253547.2000000012222461XStatistics Department, California Polytechnic State University, 1 Grand Avenue, San Luis Obispo, CA 93407 USA; 4 grid.253547.2000000012222461XDepartment of Modern Languages, California Polytechnic State University, 1 Grand Avenue, San Luis Obispo, CA 93407 USA; 5 San Luis Obispo County Women, Infants, and Children Supplemental Nutrition Program, 2191 Johnson Ave, San Luis Obispo, CA 93401 USA; 6 Santa Barbara County Women, Infants, and Children Supplemental Nutrition Program, 315 Camino del Remedio, Santa Barbara, CA 93110 USA

**Keywords:** Postpartum, WIC, Internet-based, Lifestyle intervention, Weight retention, Pregnancy

## Abstract

**Background:**

High postpartum weight retention is a strong independent risk factor for lifetime obesity, cardiovascular disease, and type 2 diabetes in women. Interventions to promote postpartum weight loss have met with some success but have been limited by high attrition. Internet-based treatment has the potential to overcome this barrier and reduce postpartum weight retention, but no study has evaluated the effects of an internet-based program to prevent high postpartum weight retention in women.

**Methods/Design:**

Fit Moms/*Mamás Activas* targets recruitment of 12 Women, Infants and Children (WIC) Supplemental Nutrition Program clinics with a total of 408 adult (>18 years), postpartum (<1 year) women with 14.5 kg or more weight retention or a body mass index of 25.0 kg/m^2^ or higher. Clinics are matched on size and randomly assigned within county to either a 12-month standard WIC intervention or to a 12-month WIC enhanced plus internet-based weight loss intervention. The intervention includes: monthly face-to-face group sessions; access to a website with weekly lessons, a web diary, instructional videos, and computer-tailored feedback; four weekly text messages; and brief reinforcement from WIC counselors. Participants are assessed at baseline, six months, and 12 months. The primary outcome is weight loss over six and 12 months; secondary outcomes include diet and physical activity behaviors, and psychosocial measures.

**Discussion:**

Fit Moms/*Mamás Activas* is the first study to empirically examine the effects of an internet-based treatment program, coupled with monthly group contact at the WIC program, designed to prevent sustained postpartum weight retention in low-income women at high risk for weight gain, obesity, and related comorbidities.

**Trial registration:**

This trial was registered with Clinicaltrials.gov (identifier: NCT01408147) on 29 July 2011.

## Background

Of the approximately four million women who give birth in the United States each year, an estimated 25% experience major weight gain after pregnancy, retaining more than 4.5 kg [1,2]. High postpartum weight retention is particularly prevalent (40 to 60%) in low income Hispanic women [3-9] and women facing food insecurity [10-13]. High postpartum weight retention is a strong independent risk factor for several long-term health consequences for the mother, including a continued trajectory of weight gain over time [14,15] and increased risk of lifetime obesity, cardiovascular disease, and type 2 diabetes. Moreover, women with high postpartum weight retention are heavier prior to their next pregnancy, which increases the risk of pregnancy-related complications, obesity, and obesity and serious health complications in the offspring [16-21]. Overweight and obese women are at high risk of postpartum weight retention and face compounded risks associated with obesity and high postpartum weight retention [22].

### Challenges in reducing postpartum weight retention

Efforts to promote postpartum weight loss in high weight retainers have met with some success and yielded short-term weight losses of 4.8 to 7.8 kg [23-26]. Lovelady *et al.* [23] promoted weight loss in newly postpartum (four weeks) overweight women with a 10-week eating and exercise program; results indicated that intervention participants lost 4.8 kg, while control participants lost 0.8 kg. Similarly, O’Toole *et al.* [25] found that a structured diet and exercise program that included weekly and bi-monthly group meetings promoted significant changes in weight and body fat relative to a self-directed control group at one-year postpartum. Leermakers *et al.* [24] designed a weekly correspondence-based behavioral intervention delivered for six months during the postpartum year in women who had retained more than 6.8 kg of their pre-pregnancy weight. Women in the correspondence group lost 7.8 kg compared to only 4.9 kg in the no-treatment control group. These weight losses compare favorably to those seen in postpartum weight loss and other behavioral programs that include weekly treatment meetings [27].

However, while effective in promoting weight loss, existing postpartum interventions have had several limitations. Interventions have been short-term (≤6 months) and generally experienced high attrition (30 to 40%). During the postpartum period, women are busy with childcare and multiple new demands, making attendance at weekly face-to-face interventions problematic [22]. Moreover, existing interventions have paid little attention to the cultural, social, and contextual factors that influence postpartum body weight [22]. Lack of a theoretical framework may underlie this shortcoming [28]. Finally, existing programs have generally failed to target a population at greatest risk for postpartum weight retention, including low-income, multiethnic women.

Lifestyle interventions during pregnancy can prevent excessive gestational weight gain and reduce postpartum weight retention in many women through six months postpartum [29-37]. However, despite intervention, the majority (50 to 70%) of women - and low-income women in particular - continue to experience high postpartum weight retention, and, by 12 months postpartum, there are no significant differences between intervention and control groups [29,30,38]. Although there are benefits to intervening during pregnancy, effective postpartum interventions are still needed to sustain positive changes instituted during pregnancy and to help women with high postpartum weight retention when pregnancy interventions appear ineffective.

### Internet-based treatment holds promise in reducing postpartum weight retention

Over the past decade, internet use has increased exponentially in the United States and most rapidly among young adult, low-income, and minority sub-groups [39-44]. In the United States, an estimated two out of three adults and 75% of adults under the age of 50 use the internet, with 88% of users having access from home [40-42,45]. Similar usage rates are prevalent among low-income women [46]. The flexibility of an internet-based weight control program could accommodate postpartum women who face many unpredictable time and childcare demands that have limited their attendance at traditional face-to-face treatment programs.

The internet has been shown to be effective in promoting significant weight loss in obese non-postpartum patient populations [47-50]. Randomized controlled trials have shown that programs delivered via the internet are effective in a variety of patient populations when they mimic standard face-to-face interventions (participants receive weekly lesson material); they submit weekly diaries and individualized email feedback is sent to the participant [47-50]. These programs have produced weight losses of 6 to 8 kg at three and six months and 4 to 8 kg at 12 [50] and 18 [47] months in non-postpartum populations. Participant retention in these programs is higher than standard face-to-face interventions, with retention rates of 80 to 84% at one year [47,50]. Less intensive internet-based weight loss programs available to the general public (for example, interactive dieting websites) that do not provide personalized feedback have had very limited effects [51,52]. However, the effectiveness and flexibility of comprehensive online behavioral treatment are potentially ideal for postpartum women who face many unpredictable time and childcare demands yet report high motivation for weight control [53-57].

### Reaching underserved postpartum women

The government-funded Women, Infants and Children (WIC) Supplemental Nutrition Program is designed to improve the nutritional status and health of low-income, nutritionally-at-risk pregnant or lactating women and children under five-years-old by providing supplementary food (in the form of coupons or checks redeemable for specific food items), nutrition education, and referrals. WIC has been in place for over four decades and serves eight million low-income women and their families each month nationwide [58]. Almost half of pregnant women in the United States and/or their children enroll in WIC at some point during their postpartum year [59]. California has the second largest WIC program in the nation, serving about 1.4 million participants monthly [60]. Evaluation research has documented improved nutritional outcomes from WIC participation, but no formal weight loss program is in place [61-65]. WIC commonly uses the internet to provide nutrition education to its patients, and patients report a preference for using the internet due to its ease and format [66]. In one of the few evaluations of a WIC website, a study of 39,541 self-selected women in the WIC program found that 75% had access to the internet from home [46]. Use of the website (was associated with significant improvements in readiness to change on a variety of domains, including picky eating, breastfeeding, and physical activity [46,66,67]. However, an internet-based intervention to reduce postpartum weight retention has neither been formally provided nor tested as part of the WIC program, where an estimated 40 to 60% of women experience high postpartum weight retention [11-13].

### Social cognitive theory

Social cognitive theory (SCT) emphasizes the dynamic interplay of the individual and the environment in adopting long-term behavior change. SCT suggests that a ‘resilient’ sense of self-efficacy must be developed to perform weight control behaviors (being physically active and eating a low-calorie diet) and needs to be developed over time through a series of mastery experiences. Those mastery experiences must promote changes in the use of self-regulation skills (planning, self-monitoring, problem solving, self-standards, goals, and self-incentives) to foster weight control and healthy eating and exercise behaviors. SCT further suggests the process of behavior change and maintenance is enhanced through social support and an awareness of the individual’s ecology. Weight control intervention does not rely solely on individual-level information, external reinforcement, or self-regulation, but it embeds a more dynamic approach to self-regulation (adaptive self-regulation skills) within the network of social influences germane to an individual’s ecology. Intrapersonal, interpersonal, and organizational and institutional supports are garnered to address particular barriers to change (such as limited time for physical activity or meal preparation, and unsafe neighborhoods) and practice changes within specific cultural contexts [68,69]. SCT thus provided a rich basis for promoting weight loss in low-income, multiethnic, postpartum women, capitalizing on the internet’s cost efficiencies, tailoring ability, ready and long-term access, and reach [70].

### Study aims

Fit Moms/*Mamás Activas* is a cluster randomized clinical trial funded by the National Institute of Diabetes, Digestive, and Kidney Diseases (NIDDK). The clinical site is at California Polytechnic State University, San Luis Obispo (S Phelan, Principal Investigator (PI)) and an additional performance site that is overseeing development and delivery of the online and text-messaging intervention is the University of North Carolina at Chapel Hill (D Tate, PI). In this cluster randomized trial, 12 WIC clinics are randomized to receive the standard WIC program for 12 months or the enhanced WIC plus internet-based postpartum weight loss program. The primary hypothesis is that the enhanced WIC plus internet postpartum weight loss program will produce significantly greater weight losses than the standard WIC at six and 12 months.

Secondary aims will determine whether the enhanced WIC plus internet postpartum weight loss program will result in greater improvements in eating and exercise behaviors (examined at 0, six, and 12 months) and psychosocial parameters (depression, social support, self-efficacy, body image, and stress) than standard care. The study will examine the proportion of women who achieve pre-pregnancy weight at six and 12 months and identify baseline characteristics that may moderate the efficacy of the intervention (such as ethnicity, socioeconomic status, pre-pregnancy weight, parity, and breastfeeding status). The study will also examine the relationship between changes in behaviors, psychosocial factors, and weight changes during the 12-month period. These evaluations will help identify the mechanisms by which the intervention produces greater success compared to the standard WIC.

## Methods/Design

### Study design

**Figure 1:**
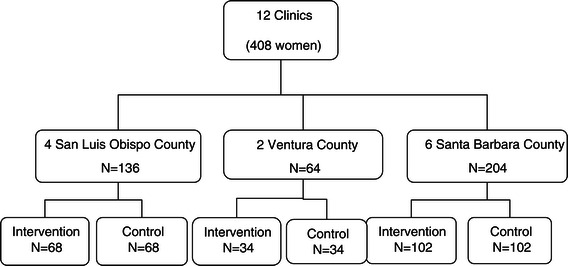
**Fit Moms/** ***Mamás Activas*** **cluster randomized design.**

As noted, Fit Moms/*Mamás Activas* is a cluster randomized (with fractional cross-over) trial evaluating the long-term effects of a comprehensive internet-based behavioral weight loss intervention for postpartum women. Twelve WIC clinics (with 408 postpartum women with excessive weight retention) are randomized within three counties to either a 12-month standard WIC intervention or to a 12-month WIC enhanced plus internet-based weight loss intervention (Figure 1). Assessments are conducted at baseline, six, and 12 months. Although the intervention’s primary target is the individual, and most of the intervention will occur outside of the clinic setting, we selected a randomized cluster design to reduce the likelihood of within-clinic contamination through patient interactions with other patients, or with WIC clinicians who are reinforcing use of the internet intervention. This study was approved by the Cal Poly Human Subjects Committee (approval number: 000001118; internet-based intervention and registered at Clinicaltrials.gov (identifier: NCT01408147). All participants in the study will provide informed, written consent using the Institutional Review Board-approved consent form.

### Clinic and participant recruitment

**Figure 2:**
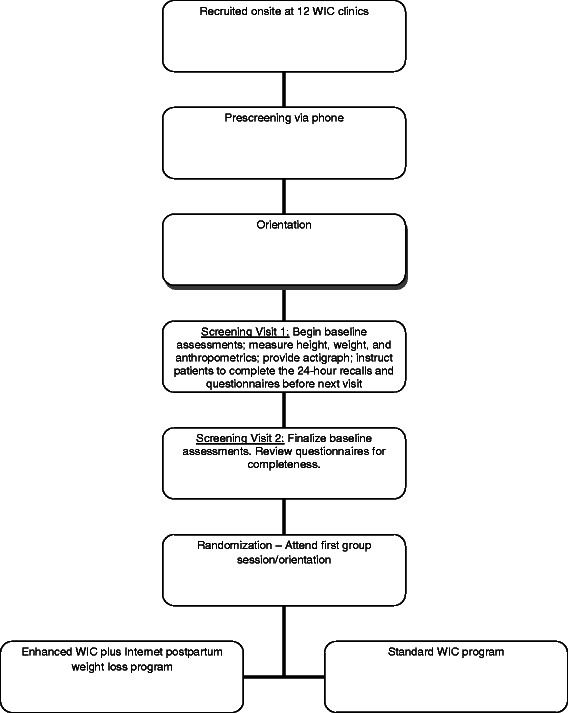
**Overview of Fit Moms/** ***Mamás Activas*** **.**

A total of 12 clinics (six in Santa Barbara county, four in San Luis Obispo county, and two in Ventura county) were selected for inclusion in this study. The clinics were selected because they are representative of California state WIC populations, are willing to accept randomization, and agreed to the study’s recruitment and intervention protocols. At these clinics, a combined total of 408 participants (34 per clinic) are targeted for recruitment. Blinded study research assistants are screening woman attending their postpartum visits, using our pre-established screener [71,72]. Women who appear interested in the program are asked to complete a permission to be contacted card and are then screened by phone; phone screening is done (rather than on-site screening) to ensure patient privacy and limit potential burden on WIC clinics of requiring private screening rooms during recruitment. If still eligible after screening, patients will be asked to attend an orientation meeting, complete informed consent, and are scheduled for baseline screening visits. Once sample sizes are met at a given clinic, recruitment is closed at the clinic. The flow chart of these stages of recruitment is shown in Figure 2.

### Eligibility criteria

Table 1 describes the eligibility and exclusion criteria for this trial. These criteria were selected to identify a representative postpartum population at greatest risk for weight gain and obesity-related co-morbidities. As indicated, women must be between six weeks and 12 months postpartum. Similar to other studies [24], we selected this period to strike a balance between allowing enough time for initial pregnancy-related weight loss to occur, while still intervening early enough to prevent long-term postpartum weight retention, which has been independently related to future weight gain and the development of obesity [15]. Women must be currently overweight or obese, with a body mass index (BMI) of 25 kg/m^2^or more or have a BMI between 22 and 24.9 and exceed pre-pregnancy weight by at least 4.5 kg (10 lb) [73]. On average, by six weeks postpartum, women retain 3 to 6.8 kg of the weight gained during pregnancy, with Hispanic populations falling into the higher end of this range [6,8]. A greater than 6.8 kg weight retention after six weeks postpartum would represent a significantly higher than average weight retention [6,8], increasing the risk of major weight gain later in life.

**Table 1:** **Inclusion and exclusion criteria for Fit Moms/** ***Mamás Activas***

**Inclusion criteria**	
	Between six weeks and 12 months postpartum
	BMI ≥25 kg/m^2^ or have a BMI between 22 and 24.9 25 kg/m^2^ and exceed pre-pregnancy weight by at least 4.5 kg (10 lb).
	18 to 40 years
	English- or Spanish-speaking
	Non-smoking
	Primiparous or multiparous
	Lactating or non-lactating
	Literacy ≥5^th^ grade level
	Cell phone
**Exclusion criteria**	
	Pregnancy or planning pregnancy
	Relocating in the next year
	Serious current physical disease (such as heart disease, cancer, renal disease, or diabetes) for which physician supervision of diet and exercise prescription is needed
	Physical problems that limit the ability to exercise
	History of eating disorders
	Current problems with drug abuse
	Current treatment for a serious psychological disorder

As indicated, women must be also be between 18 and 40-years-old; English- or Spanish-speaking; non-smoking; primiparous or multiparous; lactating or non-lactating [23,74-76]; and have adequate literacy (at a fifth grade level or beyond). Participants who do not have a computer and/or internet access from home are provided with a laptop computer and/or internet access for study use. Those with limited computer experience will receive training until they demonstrate proficiency in accessing online materials. As internet access is increasing exponentially in this country, we wanted this study’s findings to be relevant to most all populations, as internet access will likely reach the vast majority of the American population in years to come [44]. Cell phone access at time of study entry is also required. Any type of cell phone is acceptable (the phone does not have to have a smartphone). Participants whose computers or cell phones break are loaned a replacement on a temporary basis.

Exclusion criteria (Table 1) were developed to maximize the safety of the intervention and minimize attrition. These include pregnancy, planning pregnancy, relocating in the next year, serious current physical disease (such as heart disease, cancer, renal disease, or diabetes) for which physician supervision of diet and exercise prescription is needed, physical problems that limit the ability to exercise [77], a history of eating disorders [78], current problems with drug abuse, or current treatment for a serious psychological disorder (such as schizophrenia or bipolar disorder). Up to 10% of postpartum women may experience depression [79]. Women with significant depressive symptoms (Beck Depression Inventory score >20 [80-82]) require provider approval (for example, from a social worker or physician) prior to enrollment or are referred for appropriate care.

### Randomization and masking

Randomization is computer-generated by the statistician. Specifically, randomization is conducted at the clinic level, stratified by county, and designed to ensure balance in treatment arms. Four clinics were crossed-over (in random order) in order to allow for estimation of some within-clinic effects. We decided to stratify randomization by county to control for any chance imbalance due to potentially unmeasured regional or administrative differences. We decided against further stratifying based on ethnicity and pre-pregnancy BMI due to the small likelihood of a chance imbalance, given the demographic similarity of the clinics, and limited evidence that these variables impact treatment response among postpartum (or other) women [83,84]. Within each randomized clinic, participants are sequentially selected until target sample sizes are met (please see Sample size calculation section**).**

Recruiters and assessors are blinded to the clinic randomization, and participants are not told their assigned treatment status until after enrollment. Several procedures are in place to ensure blinding among clinic recruiters, assessors, and potential enrollees. In brochures and the study consent form, the study is presented identically at both control and intervention clinics. There is a minimal amount of cross-over activity between clinics, but the study’s branded items are distributed at both intervention and control clinics as part of retention so that the study logo is a familiar sight. The recruiters and assessors have little to no contact with individuals aware of treatment assignment, including the WIC county chiefs and intervention research assistants.

### Experimental conditions

#### Group one: standard Women, Infant and Children Supplemental Nutrition Program

The WIC program is a federal nutrition program for low-income women and their children that provides healthy foods, nutrition education, breastfeeding advice and support, and referrals. WIC clinics across the nation utilize a protocoled system for standardizing the frequency of contact and content of each patient-practitioner visit. At one to six weeks postpartum, women are seen by a WIC clinician, assessed, and assigned a contact code based on their breastfeeding status and health risk. The minimum frequency of contact, according to federal requirement, is five times during the first postpartum year (at six weeks, three months, six months, nine months, and 12 months) and at least four times during subsequent years (for example, 15 months, 18 months, 21 months, 24 months, and so forth). More frequent visits (one per month) may be offered during the first postpartum year to women who are at high risk (such as those who have medical problems or very limited income). Visits are conducted by nutritionists or WIC public health aides who provide nutrition counseling and referrals as needed, general support and guidance, and food vouchers. Women desiring to lose weight are advised to limit portions and increase activity; however, no structured weight control program is currently provided.

In Fit Moms/*Mamás Activas*, women randomized to standard care receive the standard WIC intervention plus a brief orientation to the study (to serve the purpose of bonding participants to the study) and study newsletters every two months with basic information about weight control, exercise, nutrition, and wellness.

#### Group 2: enhanced Women, Infant and Children Supplemental Nutrition Program plus internet-based weight control program

Patients in this group receive all elements of group one (standard WIC) plus an enhanced WIC and internet-based weight loss program. Given that it is now widely recognized that, to be effective, weight control treatment must be continued in long-term [85], we designed the intervention to continue for the duration of the trial (12 months). As noted earlier, our intervention is rooted in social learning theory and the social ecological model of behavior change and, thus, targets individual, interpersonal, and institutional levels, as outlined below.

### Individual level intervention: internet-based weight control program

#### Overview

Developed and evaluated in several interventions of up to one year by Tate *et al.* [49,50,86,87] this comprehensive, individually focused, SCT theory-driven, weight control program includes education, behavioral self-regulatory strategies, continuing contact, prompting, and access to social support. Tate *et al.* have examined this internet behavioral weight control program in several studies [50,87]. These effective online interventions are based on the Diabetes Prevention Program (DPP) and Look Ahead lifestyle interventions [88,89], which also have been proven effective in promoting long-term weight loss in multiethnic, low literacy, English- and Spanish- speaking individuals across the country [88,89]. Since the DPP and Look Ahead interventions involved individual meetings with treatment professionals, the online lessons and assignments were re-written to be more self-directed, and tailored to individuals’ needs through weekly assessments of progress upon login and programmed algorithms, rather than tailored by the human counselor. In Fit Moms/*Mamás Activas*, the intervention has been further adapted to fit needs of a postpartum, low-income, low literacy population; the website includes shorter written content, more pictures of pregnancy, mothers, and babies, and sleep and stress management components to address the challenges of caring for a baby and cooking on a budget while changing lifestyle behavior. Instructional lesson overviews and other videos provide information in a visual format.

#### Website

The website is not specific to one particular culture or dietary intake pattern, but rather attempts to provide guidance and resources that may be helpful to individuals from a variety of different cultures, incomes, and backgrounds. The website content is available in both English and Spanish and is accessible to each participant anywhere she has an internet connection. Each individual has a unique username and password. Participants can choose to log in at home, at work, or elsewhere. Patients are provided with a ‘technical assistance’ number to call should they encounter any difficulties in accessing the website. New content (lesson, tip, or link) is provided each week, but the site is designed to permit flexibility in the pace of presentation of new information. Participants are encouraged to login on Mondays to get new guidance, and then may check back periodically to see if new messages have been posted to the message board or to look for a specific link. This website was adapted from previous websites developed for research in Tate and colleagues [49,50,87] by NuMudia Productions Inc, Wake Forest, NC; USA.

#### Web Diary

Participants are encouraged to use an internet diary to look up foods and record intake. The diary (available in English or Spanish) is integrated with a comprehensive physical activity web diary. If patients are unable or unwilling to use the online diary, they are provided with paper diaries or encouraged to use alternative online tracking resources. Evidence-based strategies associated with lowering calorie intake are promoted, including following a simple, culturally relevant meal plan designed to be low cost and family-oriented.

#### Links

An organized directory of links is provided to quality diet-related sites (such as those that provide general nutrition, menu planning, recipes, and/or weight loss tips), exercise-related sites (such as those that provide lifestyle activities, information on overcoming barriers, and/or walking information), and behavioral and other sites (such as those that provide information on managing emotional eating) that are monitored by program staff for quality, relevance, and timeliness.

#### Weekly structured weight management lessons

Each lesson focuses on a topic related to weight regulation, eating, exercise, or behavior change strategies [88,89]. Each new behavioral lesson is summarized in a brief, introductory video and can also be viewed through on-screen text with visuals that may be printed for reference. Lessons are posted weekly for the intervention duration. Topics include: selecting appropriate calorie goals, modifying fat intake, increasing fiber, grocery shopping, label reading, restaurant eating, beginning exercise, aerobic fitness, self-monitoring, stimulus control, problem solving, social assertion, goal setting, body image, cognitive strategies for avoiding negative thinking and cognitive errors, overcoming barriers, relapse prevention training, strategies of successful weight losers in the National Weight Control Registry [90], and numerous other diet or exercise topics based on the DPP [84], and other weight, nutrition, and exercise projects.

#### Dietary goals

Participants are provided with sample meal plans and are instructed to follow a standard calorie restriction diet used in behavioral weight loss programs [84,88,89]. The meal plans include the option of using liquid meal replacements, but meal replacements are not provided as part of the intervention. Calorie goals are designed to produce a weekly weight loss of 0.5 to 1 kg and are based on study entry weight and breastfeeding status. Specifically, women whose current weight is under 91 kg, 91 to 115 kg, or over 115 kg are provided with 1,200 calorie per day, 1,500 calorie per day, and 1,800 calorie per day goals, respectively. Women who are exclusively breastfeeding are prescribed 300 additional calories per day. Participants are also instructed to reduce calories from fat to 35%, as calorie plus fat restriction has been shown to produce better weight loss than calorie restriction alone [91]. A diet rich in whole grains, fruits, vegetables, and lean meats or beans is encouraged, recognizing a variety of dietary choices and styles from different cultures, incomes, and backgrounds.

#### Exercise goals

Participants are instructed to gradually increase their physical activity to at least 30 minutes per day on most days of the week in moderate-intensity activities [84,88,89,92]. ‘Baby-friendly’ and inexpensive activities are suggested, taking into consideration women’s potentially unsafe neighborhoods. Walking is encouraged, but many different moderate-intensity exercises are suggested. Once participants reach 30 minutes per day on most days, they are encouraged to maintain or increase their total amount of exercise to between 60 and 90 minutes every day, as a higher level of physical activity may be necessary to achieve long-term weight loss maintenance [93-96].

#### Weight loss goals

Participants are given a scale and told to expect weight loss of 0.5 to 1 kg per week until reaching their weight loss goal. Women who are 10 pounds or more above their pre-pregnancy weight are encouraged to set an initial goal of getting back to their pre-pregnancy weight. Women who are within 10 pounds of their pre-pregnancy weight and overweight or obese are encouraged to set a 10% weight loss goal. Patients desiring to lose more weight are encouraged to do so, provided they maintain reasonable eating and activity patterns, and do not reduce below normal weight. Moderate weight loss does not appear to adversely affect lactation [23,74,75], but the study is monitoring such changes and tailoring goals accordingly.

#### Computer-tailored content and feedback

Each week upon participant login, the participant completes a check-in that inquires about weight loss progress and barriers to reaching goals. Responses are compared with pre-programmed algorithms to determine if progress is on track with expected weight loss, slow, or nonexistent. Tailored content (additional readings, links, and pdf tools), feedback, and recommendations are provided in a specific section of the website homepage based on progress and individual barriers.

#### Online message boards

From the study website, participants can access a secure, study-specific message board available in English or Spanish. The message board is designed to provide patients with opportunities for group support, problem solving, and feedback from study interventionists and peers. At any time, participants may post questions or comments. The board is moderated on a daily basis by study interventionists, who answer questions and provide feedback.

#### Instructional and inspirational videos

As noted, each behavioral lesson includes an introductory video that summarizes the lesson. Additional instructional videos review label reading, portion sizes, self-monitoring, and other key tools for successful weight control. An inspirational video series documenting the journeys of two women from the population engaging in postpartum weight loss is also included on the website. These women, fluent in both English and Spanish, volunteered to be profiled as part of a video blog in a pilot study and featured on the site. Other entertainment, unrelated to weight loss (such as a telenovela type of soap opera series), is provided for the first six months as a means of attracting women to logon more frequently.

#### Text messaging

Each week, participants receive four SMS messages at random days and times. The messages are designed to reinforce use of the online weight loss program and provide tips, suggestions, positive reinforcement, and encouragement for weight control behaviors, using a protocol similar to one tested effectively in other studies [97]. Messages are in four domains: 1) related to weekly content on the site, 2) motivation, 3) behavioral skills, and 4) individualized feedback based on participant progress. Patients are not asked to respond to the text messages.

### Institutional and interpersonal level intervention

#### Brief clinic visits

WIC public health aides and dietitians spend five minutes during patients’ regularly scheduled WIC visits (typically every three months) to reinforce use of the internet-based program. After WIC practitioners logon to the WIC check-in system, they receive an alert and instructions to provide the patient with a specific Fit Moms/*Mamás Activas* color-coded card, tailored to each patient’s level of adherence to logging on to the website and attending groups. The WIC counselors review the cards and encourage patients to use the Fit Moms/*Mamás Activas* site and to attend groups. We considered allocating a greater role for the WIC practitioners at these visits (such as discussing weight loss progress, goal setting, problem solving, and motivation interviewing). However, the scripted message and simplicity of the protocol were praised by the WIC staff during our design phase and have been designed to fit into the busy WIC practice setting, provide institutional-level support for the intervention, and enable delivery by both public health aides and dietitians. More extensive professional support is provided by study interventionists at monthly groups.

#### Monthly group meetings

Group meetings introduce new weight loss topics, further reinforce messages of the online program, provide opportunities to problem-solve barriers to weight loss, and provide additional support and education on selected topics, following structured protocols adapted from other studies [88,89]. Consistent with WIC practices, the groups last 60 minutes, are offered in Spanish and English, and allow adequate space for infants. The group visits are ‘closed’ to new participants and tied to receipt of WIC vouchers whenever feasible [98]. We considered having WIC dietitians lead these groups, but decided that future research will test this program as fully disseminated into the WIC program; thus, we used study staff to deliver intervention.

#### Reinforcing adherence

Adherence to behavioral goals is reinforced through allocation of ‘diaper points’. Women earn diaper points for logging onto the website, entering their weight onto the website, self-monitoring eating and exercise, posting a message on the message board, attending group sessions, and/or turning in the Fit Moms/*Mamás Activas* message cards provided by their WIC counselors. A maximum of 90 to 100 points are earnable by participants each month. Items vary in point values. For example, a package of diapers costs 75 points and wipes cost 50 points. Participants may redeem these points on the website or at a group meeting to obtain diapers or other tangible incentives.

### Outcome measures

Assessments are conducted by blinded, bilingual assessors at baseline and after six and 12 months. Questionnaires were selected based on their prior validity and use in English- and Spanish- speaking, postpartum populations. Participants are provided with a US$25 honorarium for the baseline and six-month assessments and US$50 for the 12-month visit.

### Anthropometric measures

At each assessment visit, weight is measured to the nearest 0.1 kg using a calibrated standard digital scale (Tanita Health Equipment, Arlington Heights, Illinois, USA**).** Two measures are completed with participants measured in light clothing (without shoes). Scale calibration is checked weekly with known weights. Standing height is measured twice in patients without shoes in millimeters with a calibrated portable SECA 217 stadiometer (SECA, Birmingham, United Kingdom) at WIC clinics. Waist circumference is measured at the midpoint between highest point of iliac crest and lowest point of costal margin using a Singer Measuring tape (Singer, LaVergne, TN, USA). Two measures of waist circumference are taken and, if the difference exceeds 1.0 cm, a third measure is taken. Pre-pregnancy weight is based on self-report at time of last menstrual period. Our research and other studies have suggested that women are quite accurate in recalling their pre-pregnancy weight [99,100]. However, to increase the validity, we are abstracting pre-pregnancy measured weights from clinic charts whenever available.

### Behavioral measures

#### Dietary intake

Dietary intake is assessed at baseline, six months, and 12 months using 24-hour recalls on two random days over a week [101-103], and completed using the NCI Automated Self-Administered 24-hour recall ((ASA24). The primary variables of interest are: calories, protein, carbohydrates, and fat; consumption of sugar-sweetened beverages; and consumption of fast food. Under-reporting will be estimated by comparing reported energy intakes (based on 24-hour recalls) with estimated total energy expenditure (TEE) for pregnant women [104]. If indicated, analyses will exclude extreme under-reporters. Fast food consumption is also assessed based on self-report questions used in our previous research [105].

#### Physical activity

The ActiGraph GT3X+ accelerometer (The ActiGraph, Pensacola, FL; USA) is used to measure maternal activity [106-108]. The monitor records time-varying accelerations ranging in magnitude from +/− 6 g’s. The accelerometer output is sampled by a 12-bit analog to digital convertor (The ActiGraph, Pensacola, FL; USA) and the raw acceleration is then stored in the flash memory for future analysis. The GT3X+ is small and lightweight (19 g) and is worn at the waist. The seven-day physical activity recall (PAR) is also administered as an additional measure of physical activity. The PAR is a semi-structured interview that estimates an individual’s time spent in physical activity, strength, and flexibility activities for the seven days prior to the interview [109]. Television habits and sedentary behaviors are also assessed by pre-established questionnaires [110].

#### Weight control practices

The 26-item Eating Behavior Inventory (EBI) [111,112] is used to assess the extent to which participants adhere to behavioral weight control strategies (such as stimulus control and calorie counting). In another questionnaire, self-weighing, self-monitoring, stimulus control, and problem solving are also assessed [113].

### Psychosocial measures

#### Eating inventory

The Eating Inventory (TFEQ) is a 51-item self-report instrument with three factors assessing dietary restraint, disinhibition, and hunger. The restraint factor assesses the degree of conscious control one is exerting over eating behaviors; the disinhibition factor measures susceptibility to loss of control over eating; and the hunger factor assesses hunger.

#### Home environment

Food storage in the home is assessed by having participants complete a checklist of the possible foods stored in their refrigerator and cabinets; this checklist has been used in diverse patient populations and settings [114,115]. Responses may be grouped into categories of high- and low-fat foods. The questionnaire also asks participants to record whether food items are stored in visible areas (for example, on countertops or coffee tables) within the home. The Exercise Environment Questionnaire is used to assess the amount and type of exercise equipment and televisions available in the home [116].

#### Depression

The 10-item Edinburgh postnatal depression scale is used to measure levels of depression; a score of 13 or more is used as the cutoff for probable depression [117].

#### Sleep

The ActiGraph GT3X+ accelerometer is also used to measure sleep objectively [106-108]. Sleep quality is assessed using the General Sleep Disturbance questionnaire [118].

#### Eating disorders

Items from the Eating Disorders Examination-Questionnaire (EDE-Q) [119] is used to assess the frequency of unsafe dieting practices. During the trial, participants who endorse eating disorder symptomatology (self-induced vomiting, excessive exercise, and/or use of laxatives or diuretic use for the purposes of weight loss) will be referred for appropriate evaluation and/or care, and intervention activities will cease until approved by a provider.

#### Body image

Body image is assessed using the short version of the Body Shape Questionnaire, which is a 14-item scale that measures concerns about body shape and feelings of fatness [120,121].

#### Self-efficacy

The self-efficacy subscale of the Kendall *et al.* attitudes questionnaire [122] is used to measure confidence in ability to lose weight, eat healthy, and exercise after pregnancy.

#### Stress

Perceived stress is measured using the 10-item perceived stress scale [123].

#### Social support

Social support is measured using the six-item scale of postpartum social support [124], which focuses on instrumental (such as help with caring for the baby) and emotional (tell you that you are doing a good job as a mother) support.

### Supporting measures

At baseline, participants complete a demographic questionnaire assessing age, race, ethnicity, history of intentional weight loss, and weight history. Literacy is assessed using the Newest Vital Sign [125]. Information on food security and acculturation, parity, and intervals between pregnancies are also collected to assess any prior relationship with postpartum weight retention [126-129]. Information on changes in smoking, prescription medications, unsafe dieting practices [78], job status, and participation in other weight loss programs are assessed at follow-up assessments. Participants are also asked at each follow-up assessment to report all prescription and non-prescription medication, and to indicate any health problems that they have experienced since the last assessment. Breastfeeding intensity and duration [2], and information about introduction of formula, are also assessed at each visit. Similar to our prior research, safety assessments include injuries due to physical activity, instances of extreme hunger, fatigue, and changes in milk supply.

#### Weight status of family

Based on the recognition that maternal eating, activity, and weight may influence family health, participants are asked to indicate the weight status of their partner and children in the home. As part of an ancillary study, participants may choose to have their infant’s weight, length, and skinfold objectively measured.

#### Assessment of recruitment, attrition, and referral rates

Women who decline to participate are asked their reasons for declining participation in order to inform generalizability and better understand the characteristics of individuals who choose not to participate. We are also tracking eligibility and reasons for exclusion, numbers referred to other services (psychological and so forth), drop outs, and reasons for drop out.

#### Treatment fidelity and process

Research assistants are tracking the number of newsletters sent. At the 12-month assessment, participants are also asked to report on the frequency they received and read newsletters; they also complete a questionnaire to rate the usefulness and satisfaction with Fit Moms/*Mamás Activas* content and its delivery, which includes ratings of various aspects of the program. In the intervention group, data (via background website monitoring) are also available on the number of logins and number of online self-monitoring records completed, frequency of posting to the message boards, viewing of video resources, redemption of diaper points, and page views for various website features. Attendance at monthly group meetings is also tracked. All group treatment sessions are audiotaped, and a random 20% of these sessions are being reviewed by an interventionist who did not conduct the intervention session. The reviewer listens to the session tape and completes a fidelity checklist developed specifically for this trial to confirm that the essential content for each session is being covered.

#### Cluster-level assessment

Basic demographic information and lactation status is collected in all women in the WIC. In addition, we are assessing any changes to each clinic’s nutrition and physical activity programs and documenting any external independent programmatic policy and environmental changes. Data are collected annually related to levels of physical activity and nutrition that may affect women in the WIC, including: (1) aspects of the environment in the clinic and surrounding neighborhood (such as the promotion of walking programs); (2) relevant grants and research program initiatives at clinics; (3) local, state, or federal mandates; and (4) promotions and advertising.

#### Procedures to retain sample

Clinic retention strategies include maintaining ongoing contact with state, county, and local WIC agencies. At the local level, we provide small incentives and ‘lunch and learn’ sessions to update clinics on study progress. Participant retention strategies have been used effectively in our other studies of multiethnic childbearing women, and include collecting multiple phone numbers and numbers of relatives and friends for contact. For each data collection visit, participants have their visit scheduled by phone, are sent written reminders, and are called the day before. Missed visits are rescheduled and followed up on. Costs for transportation and childcare coverage are provided to participants with repeat missed assessments. If necessary, assessments are completed at patients’ homes. Compensation is provided to further promote retention: US$25 for completing the baseline and six-month assessments and US$50 for the 12-month assessment.

#### Sample size calculation

The targeted sample size (12 clinics; 34 participants per clinic; total N = 408) is projected to provide ≥80% power to detect a meaningful difference in weight change at 12 months as small as 2 kg between group means, when the standard deviation is 4.5 kg and the intraclass correlation (ICC) is 0.02, using a two-sided *t*-test with a significance level of 0.05, and accommodating anticipated rates of loss to follow-up (30% participant attrition at 12 months). Although we do not anticipate clinic attrition, 10 clinics (five in each condition) would provide 80% power to detect a difference in weight change as small as 2 kg. An ICC of 0.002 was estimated from results of our pilot demonstration trial and a WIC survey study [130], but we have conservatively used an ICC of 0.02 for sample size calculations [131]. For secondary aims, in our most conservative estimates [25], we would have greater than 80% power to detect both medium and large effects (Cohen’s d = 0.5 and 0.8, respectively) for any continuous or categorical outcome variable at the alpha = 0.05 level using a two-sided test of significance.

#### Analysis plan

The primary outcome measure, change in weight from baseline over time, is assessed at six and 12 months. All measured weights will be included in the analyses. The analysis strategy is based on a conceptual framework that explicitly recognizes that both differences between individuals within clinics and between clinics may contribute to variance in weight change. Randomly assigned group (standard care versus intervention) is the main independent variable for intention-to-treat (ITT) analyses. County is a stratification variable for randomization and is expected to be balanced across intervention and standard care groups by design. Our analysis approach uses linear mixed-effect models to analyze differences between the intervention and standard care clinics allowing for within-cluster variation (participants within the same clinic) and within-individual variation (repeated measures) [132]. The model will include factors for a fixed effect for treatment and group and random effects for clinic as well as a group × time interaction term to test if the change over time in the dependent variable differs significantly for the two study groups. In addition, for primary and secondary outcomes, the model will include participant-level covariates to adjust for any baseline differences, or adjust for covariates that may relate to the outcome, including lactation, age, age at menarche, education, income, smoking status, language, socioeconomic status, race and ethnicity, height, pre-pregnancy BMI, parity, gestational gain, inter-pregnancy interval, baseline BMI, and changes in marital status and employment [2,133-139] In addition, the model will include terms to account for whole-clinic (cluster-level) variants, including Latina proportion, lactation proportion, mean age, mean education, and mean SES.

Our study design will give rise to temporally repeated measurements, and some subjects may be lost to follow up. In the primary analysis, missing weight measurements will be assumed missing at random, but diagnostic analyses will be conducted related to missing data (for example, comparing baseline characteristics of completers versus non-completers and creating propensity scores). If missing data appear to have the potential of influencing interpretation, we will also conduct multiple imputations using several different models for non-ignorable missingness. These will be used to assess the range of impact that missing data may exert on our results.

A secondary aim is to test the hypothesis that the intervention will result in a greater percentage of the women achieving their preconception weight or below at 12 months. For this analysis, a generalized linear mixed-model (binomial with a logit link) will be used for proportion at preconception weight or below at six and 12 months postpartum, including the same predictors described above. For secondary analyses comparing changes in behavioral and psychosocial variables, the effect of treatment at each time point will be investigated using the same basic linear mixed-effects model described above. In analyses of the possible differential effects of treatment on various subgroups of women, interactions between patient-level covariates (as in the main analyses) and group will be examined. A Bonferroni adjustment will be used to adjust for multiple comparisons. Also, generalized linear mixed-model analyses, as described above, will also be conducted to examine whether baseline variables moderate weight loss outcomes. Analyses of whether any effect of the intervention is mediated by differences in demographic, behavioral, and psychosocial factors will be conducted by first eliminating variables that have no impact on weight loss, then variables of interest will be entered into a final linear mixed-effects model, using both theoretical considerations to guide variable selection and the statistical strength of potential predictor variables [140].

## Discussion

Several researchers [22,28,141-146] and governmental bodies [147,148] have called for theory-driven empirical studies evaluating interventions that occur after pregnancy, with the specific aim of preventing postpartum weight retention and the diseases that follow. The proposed intervention is one of the first to apply SCT in targeting both individual and institutional levels of support. The intervention is further designed to capitalize on a natural period of redefinition that occurs among women after pregnancy [54,149,150], paying special attention to several unique physiological, psychological, cultural, and lifestyle changes that occur postpartum.

Internet-based behavioral treatment has been effective in promoting weight loss in other populations, but there have been no studies of internet weight control programs for postpartum women. Since postpartum women are busy with childcare and multiple new demands, a flexible treatment approach is likely needed [22]. The distantly delivered nature of the Fit Moms/*Mamás Activas* intervention, coupled with its integration within the WIC, have the potential to positively impact the large number of American women who enroll in the WIC and experience high postpartum weight retention, yet have limited access to effective weight loss treatment.

We are proposing to first evaluate the efficacy of the Fit Moms/*Mamás Activas* intervention to reduce postpartum weight retention in a controlled fashion. We are testing the effects of an intervention package that targets individual, interpersonal, and institutional levels. If the intervention is found to be efficacious, future research may disentangle the relative contribution of each of these levels in reducing postpartum weight retention. Findings from the current study will also inform further research in the costs and effectiveness of this program, as implemented by the WIC, and the best methods for sustaining and disseminating the treatment on a more global basis to other WIC populations. We are partnering with the State of California WIC program in mutual agreement that the proposed intervention could be effectively disseminated into WIC programs in the state of California. We believe the intervention in the proposed study will ultimately provide a much-needed service to improve the long-term health status of underserved women of reproductive age in the state of California and beyond. A lifestyle intervention that reduces postpartum weight retention in low-income women has the potential to shift current treatment practices in the WIC and impact the health of many low-income women in the United States.

### Trial status

This study is currently recruiting participants. Recruitment began and is expected to end December, 2015.

## Abbreviations

WIC: Women, Infants, Children Nutrition Program
SCT: Social cognitive theory
NIDDK: National Institute of Diabetes, Digestive, and Kidney Diseases
DPP: Diabetes Prevention Program)
TEE: Total energy expenditure)
PAR: Physical activity recall)
TFEQ: Three factor eating questionnaire/eating inventory
EDE-Q: Eating disorders examination-questionnaire)
ITT: Intent-to-treat)
ICC: Intraclass correlation coefficient

## References

[1] Keppel KG, Taffel SM (1993). Pregnancy-related weight gain and retention: implications of the 1990 Institute of Medicine Guidelines. Am J Public Health.

[2] Ohlin A, Rossner S (1990). Maternal body weight development after pregnancy. Int J Obes.

[3] Parker JD, Abrams B (1993). Differences in postpartum weight retention between black and white mothers. Obstet Gynecol.

[4] Walker LO, Timmerman GM, Sterling BS, Kim M, Dickson P (2004). Keeping pregnancy-related weight may result in long-term weight problems for women. Ethn Dis.

[5] Kac G, Benicio MH, Velasquez-Melendez G, Valente JG (2004). Nine months postpartum weight retention predictors for Brazilian women. Public Health Nutr.

[6] Walker LO, Sterling BS, Timmerman GM (2005). Retention of pregnancy-related weight in the early postpartum period: implications for women’s health services. J Obstet Gynecol Neonatal Nurs.

[7] Walker L, Freeland-Graves JH, Milani T, George G, Hanss-Nuss H, Kim M (2004). Weight and behavioral and psychosocial factors among ethnically diverse, low-income women after childbirth: II. Trends and correlates. Women Health.

[8] Walker LO (2007). Managing excessive weight gain during pregnancy and the postpartum period. J Obstet Gynecol Neonatal Nurs.

[9] Schauberger CW, Rooney BL, Brimer LM (1992). Factors that influence weight loss in the puerperium. Obstet Gynecol.

[10] Olson CM, Strawderman MS (2008). The relationship between food insecurity and obesity in rural childbearing women. J Rural Health.

[11] Schieve LA, Cogswell ME, Scanlon KS (1998). Trends in pregnancy weight gain within and outside ranges recommended by the Institute of Medicine in a WIC population. Matern Child Health J.

[12] Ostbye T, Krause KM, Swamy GK, Lovelady CA (2010). Effect of breastfeeding on weight retention from one pregnancy to the next: results from the North Carolina WIC program. Prev Med.

[13] Krause KM, Lovelady CA, Peterson BL, Chowdhury N, Ostbye T (2010). Effect of breast-feeding on weight retention at 3 and 6 months postpartum: data from the North Carolina WIC Programme. Public Health Nutr.

[14] Rooney BL, Schauberger CW (2002). Excess pregnancy weight gain and long-term obesity: one decade later. Obstet Gynecol.

[15] Linne Y, Dye L, Barkeling B, Rossner S (2004). Long-term weight development in women: a 15-year follow-up of the effects of pregnancy. Obes Res.

[16] Villamor E, Cnattingius S (2006). Interpregnancy weight change and risk of adverse pregnancy outcomes: a population-based study. Lancet.

[17] Driul L, Cacciaguerra G, Citossi A, Martina MD, Peressini L, Marchesoni D (2008). Prepregnancy body mass index and adverse pregnancy outcomes. Arch Gynecol Obstet.

[18] Samuels-Kalow ME, Funai EF, Buhimschi C, Norwitz E, Perrin M, Calderon-Margalit R (2007). Prepregnancy body mass index, hypertensive disorders of pregnancy, and long-term maternal mortality. Am J Obstet Gynecol.

[19] Boney CM, Verma A, Tucker R, Vohr BR (2005). Metabolic syndrome in childhood: association with birth weight, maternal obesity, and gestational diabetes mellitus. Pediatrics.

[20] Watkins ML, Botto LD (2001). Maternal prepregnancy weight and congenital heart defects in offspring. Epidemiology.

[21] Olson CM, Strawderman MS, Dennison BA (2009). Maternal weight gain during pregnancy and child weight at age 3 years. Matern Child Health J.

[22] Gore SA, Brown DM, West DS (2003). The role of postpartum weight retention in obesity among women: a review of the evidence. Ann Behav Med.

[23] Lovelady CA, Garner KE, Moreno KL, Williams JP (2000). The effect of weight loss in overweight, lactating women on the growth of their infants. N Engl J Med.

[24] Leermakers EA, Anglin K, Wing RR (1998). Reducing postpartum weight retention through a correspondence intervention. Int J Obes.

[25] O’Toole ML, Sawicki MA, Artal R (2003). Structured diet and physical activity prevent postpartum weight retention. J Womens Health (Larchmt).

[26] Kinnunen TI, Pasanen M, Aittasalo M, Fogelholm M, Weiderpass E, Luoto R (2007). Reducing postpartum weight retention–a pilot trial in primary health care. Nutr J.

[27] Wing RR, Wadden TA, Stunkard AJ (2002). Behavioral weight control. Handbook of obesity treatment.

[28] Keller C, Records K, Ainsworth B, Permana P, Coonrod DV (2008). Interventions for weight management in postpartum women. J Obstet Gynecol Neonatal Nurs.

[29] Polley BA, Wing RR, Sims CJ (2002). Randomized controlled trial to prevent excessive weight gain in pregnant women. Int J Obes Relat Metab Disord.

[30] Olson CM, Strawderman MS, Reed RG (2004). Efficacy of an intervention to prevent excessive gestational weight gain. Am J Obstet Gynecol.

[31] Wolff S, Legarth J, Vangsgaard K, Toubro S, Astrup A (2008). A randomized trial of the effects of dietary counseling on gestational weight gain and glucose metabolism in obese pregnant women. Int J Obes (Lond).

[32] Thornton YS (2009). Preventing excessive weight gain during pregnancy through dietary and lifestyle counseling: a randomized controlled trial. Obstet Gynecol.

[33] Shirazian T, Monteith S, Friedman F, Rebarber A (2010). Lifestyle modification program decreases pregnancy weight gain in obese women. Am J Perinatol.

[34] Claesson IM, Sydsjo G, Brynhildsen J, Cedergren M, Jeppsson A, Nystrom F (2008). Weight gain restriction for obese pregnant women: a case–control intervention study. BJOG.

[35] Artal R, Catanzaro RB, Gavard JA, Mostello DJ, Friganza JC (2007). A lifestyle intervention of weight-gain restriction: diet and exercise in obese women with gestational diabetes mellitus. Appl Physiol Nutr Metab.

[36] Mottola MF, Giroux I, Gratton R, Hammond JA, Hanley A, Harris S (2010). Nutrition and exercise prevents excess weight gain in overweight pregnant women. Med Sci Sports Exerc.

[37] Algert S, Shragg P, Hollingswroth DR (1985). Moderate caloric restriction in obese women with gestational diabetes. Obstet Gynecol.

[38] Phelan S, Phipps MG, Abrams B, Darroch F, Grantham K, Schaffner A (2014). Does behavioral intervention in pregnancy reduce postpartum weight retention? Twelve-month outcomes of the Fit for Delivery randomized trial. Am J Clin Nutr.

[39] Department of Health and Human Services, Office of Disease Prevention and Health Promotion (2000). Healthy people 2010, 2nd ed.

[40] Harwood P, Rainie L (2004). People who use the internet away form home and work (Internet.

[41] Pew Internet and American Life Project, editor (2004). Older Americans and the Internet.

[42] Cooper KB, Gallagher MD (2004). A nation online: entering the broadband age.

[43] Pew Internet and American Life Project, editor (2005). Health information online: eight in ten internet users have looked for health information online, with increased interest in diet, fitness, drugs, health insurance, experimental treatment, and particular doctors and hospitals.

[44] McNeely JK, Hoffman GM, Eckert JE (1991). Postoperative pain relief in children from the parascalene injection technique. Reg Anesth.

[45] Census Bureau US (2013). Computer and internet use in the United States.

[46] Bensley RJ, Brusk JJ, Anderson JV, Mercer N, Rivas J, Broadbent LN (2006). wichealth.org: impact of a stages of change-based internet nutrition education program. J Nutr Educ Behav.

[47] Harvey-Berino J, Pintauro S, Buzzell P, Gold EC (2004). Effect of internet support on the long-term maintenance of weight loss. Obes Res.

[48] Micco N, Gold B, Buzzell P, Leonard H, Pintauro S, Harvey-Berino J (2007). Minimal in-person support as an adjunct to internet obesity treatment. Ann Behav Med.

[49] Tate DF, Jackvony EH, Wing RR (2006). A randomized trial comparing human e-mail counseling, computer-automated tailored counseling, and no counseling in an Internet weight loss program. Arch Intern Med.

[50] Tate DF, Jackvony EH, Wing RR (2003). Effects of Internet behavioral counseling on weight loss in adults at risk for type 2 diabetes: a randomized trial. JAMA.

[51] Womble LG, Wadden TA, McGuckin BG, Sargent SL, Rothman RA, Krauthamer-Ewing ES (2004). A randomized controlled trial of a commercial internet weight loss program. Obes Res.

[52] McConnon A, Kirk SF, Cockroft JE, Harvey EL, Greenwood DC, Thomas JD (2007). The Internet for weight control in an obese sample: results of a randomised controlled trial. BMC Health Serv Res.

[53] Heneghan AM, Mercer M, DeLeone NL (2004). Will mothers discuss parenting stress and depressive symptoms with their child’s pediatrician?. Pediatrics.

[54] Fidanza AA, Simonetti MS, Cucchia LM (1986). A nutrition study involving a group of pregnant women in Assisi, Italy. Part 2: determination of vitamin nutriture. Int J Vitam Nutr Res.

[55] Baker CW, Carter AS, Cohen LR, Brownell KD (1999). Eating attitudes and behaviors in pregnancy and postpartum: global stability versus specific transitions. Ann Behav Med.

[56] Fidanza AA, Fidanza R (1986). A nutrition study involving a group of pregnant women in Assisi, Italy. Part 1: anthropometry, dietary intake and nutrition knowledge, practices and attitudes. Int J Vitam Nutr Res.

[57] Abraham S (1989). Problems with weight control during pregnancy. Med J Aust.

[58] United States Department of Agriculture (2008). WIC the special supplemental nutrition program for women, infants, and children.

[59] Cole N (2001). The prevalence of overweight among WIC children WIC-01-PCOM.

[60] California WIC Association (2008). California WIC facts.

[61] Rush D, Leighton J, Sloan NL, Alvir JM, Horvitz DG, Seaver WB (1988). The National WIC evaluation: evaluation of the Special Supplemental Food Program for Women, Infants, and Children. VI. Study of infants and children. Am J Clin Nutr.

[62] Rush D, Sloan NL, Leighton J, Alvir JM, Horvitz DG, Seaver WB (1988). The National WIC Evaluation: evaluation of the special supplemental food program for Women, Infants, and Children. V. Longitudinal study of pregnant women. Am J Clin Nutr.

[63] Rush D, Horvitz DG, Seaver WB, Leighton J, Sloan NL, Johnson SS (1988). The National WIC Evaluation: evaluation of the special supplemental food program for Women, Infants, and Children. IV. Study methodology and sample characteristics in the longitudinal study of pregnant women, the study of children, and the food expenditures study. Am J Clin Nutr.

[64] Owen AL, Owen GM (1997). Twenty years of WIC: a review of some effects of the program. J Am Diet Assoc.

[65] Caan B, Horgen DM, Margen S, King JC, Jewell NP (1987). Benefits associated with WIC supplemental feeding during the interpregnancy interval. Am J Clin Nutr.

[66] Birkett D, Johnson D, Thompson JR, Oberg D (2004). Reaching low-income families: focus group results provide direction for a behavioral approach to WIC services. J Am Diet Assoc.

[67] Bensley RJ, Mercer N, Brusk JJ, Underhile R, Rivas J, Anderson J (2004). The eHealth behavior management model: a stage-based approach to behavior change and management. Prev Chronic Dis.

[68] Whitehead M, Dahlgren G (1991). What can be done about inequalities in health?. Lancet.

[69] Committee on Assuring the Health of the Public in the 21st Century (2002). The future of the public’s health in the 21st century.

[70] Winett RA, Tate DF, Anderson ES, Wojcik JR, Winett SG (2005). Long-term weight gain prevention: a theoretically based Internet approach. Prev Med.

[71] Arozullah AM, Khuri SF, Henderson WG, Daley J (2001). Development and validation of a multifactorial risk index for predicting postoperative pneumonia after major noncardiac surgery. Ann Intern Med.

[72] Lee SY, Bender DE, Ruiz RE, Cho YI (2006). Development of an easy-to-use Spanish Health Literacy test. Health Serv Res.

[73] Lee CF, Hwang FM, Liou YM, Chien LY (2011). A preliminary study on the pattern of weight change from pregnancy to 6 months postpartum: a latent growth model approach. Int J Obes (Lond).

[74] McCrory MA, Nommsen-Rivers LA, Mole PA, Lonnerdal B, Dewey KG (1999). Randomized trial of the short-term effects of dieting compared with dieting plus aerobic exercise on lactation performance. Am J Clin Nutr.

[75] Larson-Meyer DE (2002). Effect of postpartum exercise on mothers and their offspring: a review of the literature. Obes Res.

[76] Amorim Adeqboye AR, Linne YM (2013). Diet or exercise, or both, for weight reduction in women after childbirth. Cochrane Database Syst Rev.

[77] Thomas S, Reading J, Shephard RJ (1992). Revision of the Physical Activity Readiness Questionnaire (PAR-Q). Can J Appl Sport Sci.

[78] Cooper Z, Fairburn CG (1987). The Eating Disorder Examination: a semi-structured interview for the assessment of the specific psychopathology of eating disorders. Int J Eat Disord.

[79] Mayberry LJ, Horowitz JA, Declercq E (2007). Depression symptom prevalence and demographic risk factors among U.S. women during the first 2 years postpartum. J Obstet Gynecol Neonatal Nurs.

[80] Beck AT, Steer RA (1987). Manual for the Beck depression inventory.

[81] Wiebe JS, Penley JA (2005). A psychometric comparison of the Beck Depression Inventory-II in English and Spanish. Psychol Assess.

[82] Beck AT, Steer RA (1987). Beck depression inventory.

[83] Gunderson EP, Abrams B, Selvin S (2001). Does the pattern of postpartum weight change differ according to pregravid body size?. Int J Obes Relat Metab Disord.

[84] Diabetes Prevention Program Research Group (2002). Reduction in the incidence of type 2 diabetes with lifestyle intervention or metformin. New Engl J Med.

[85] Perri MG, Corsica JA, Wadden T, Stunkard A (2002). Improving the maintenance of weight lost in behavioral treatment of obesity. Treatment of obesity.

[86] Wing RR, Tate DF, Gorin AA, Raynor HA, Fava JL (2006). A self-regulation program for maintenance of weight loss. N Engl J Med.

[87] Tate DF, Wing RR, Winett RA (2001). Using internet technology to deliver a behavioral weight loss program. JAMA.

[88] Diabetes Prevention Program (DPP) Research Group (2002). The Diabetes Prevention Program (DPP): description of lifestyle intervention. Diabetes Care.

[89] Ryan DH, Espeland MA, Foster GD, Haffner SM, Hubbard VS, Johnson KC (2003). Look AHEAD (Action for Health in Diabetes): design and methods for a clinical trial of weight loss for the prevention of cardiovascular disease in type 2 diabetes. Control Clin Trials.

[90] Wing RR, Phelan S (2005). Long-term weight loss maintenance. Am J Clin Nutr.

[91] NHLBI Obesity Education Initiative Expert Panel on the Identification EaToOaOiA (1998). Clinical guidelines on the identification, evaluation, and treatment of overweight and obesity in adults-The evidence report. National Institutes of Health. Obes Res.

[92] Jakicic JM, Wing RR, Butler BA, Robertson RJ (1995). Prescribing exercise in multiple short bouts versus one continuous bout: effects on adherence, cardiorespiratory fitness, and weight loss in overweight women. Int J Obes.

[93] Phelan S, Roberts M, Lang W, Wing RR (2007). Empirical evaluation of physical activity recommendations for weight control in women. Med Sci Sports Exerc.

[94] Pronk NP, Wing RR (1994). Physical activity and long-term maintenance of weight loss. Obes Res.

[95] Wing RR (1999). Physical activity in the treatment of the adulthood overweight and obesity: current evidence and research issues. Med Sci Sports Exer.

[96] Thomas JG, Bond DS, Phelan S, Hill JO, Wing RR (2014). Weight-loss maintenance for 10 years in the National Weight Control Registry. Am J Prev Med.

[97] Patrick K, Raab F, Adams MA, Dillon L, Zabinski M, Rock CL (2009). A text message-based intervention for weight loss: randomized controlled trial. J Med Internet Res.

[98] Carr A (2009). Breastfeeding and the WIC program. Breastfeed Med.

[99] Stevens-Simon C, McAnarney E, Coulter M (1986). How accurately do pregnant adolescents estimate their weight prior to pregnancy?. Adolesc Health Care.

[100] Phelan S, Phipps MG, Abrams B, Darroch FE, Wing RR (2011). Randomized trial of a behavioral intervention to prevent excessive gestational weight gain: the fit for delivery study. Am J Clin Nutr.

[101] Stunkard AJ, Waxman M (1981). Accuracy of self-reports of food intake: a review of the literature and a report of a small series. J Am Diet Assoc.

[102] Eck L, Klesges RC, Hanson CL, Slawson D, Lavasque ME (1991). Measuring short-term dietary intake: development and testing of a 1-week food frequency questionnaire. J Am Diet Assoc.

[103] Schaffer DM, Velie EM, Shaw GM, Todoroff KP (1998). Energy and nutrient intakes and health practices of Latinas and white non-Latinas in the 3 months before pregnancy. J Am Diet Assoc.

[104] Forsum E, Lof M (2007). Energy metabolism during human pregnancy. Annu Rev Nutr.

[105] Phelan S, Wyatt H, Nassery S, Dibello J, Fava JL, Hill JO (2007). Three-year weight change in successful weight losers who lost weight on a low-carbohydrate diet. Obesity (Silver Spring).

[106] Nichols JF, Morgan CG, Chabot LE, Sallis JF, Calfas KJ (2000). Assessment of physical activity with the Computer Science and Applications, Inc., accelerometer: laboratory versus field validation. Res Q Exerc Sport.

[107] Sirard JR, Melanson EL, Li L, Freedson PS (2000). Field evaluation of the Computer Science and Applications. Inc. physical activity monitor. Med Sci Sports Exerc.

[108] Freedson PS, Melanson E, Sirard J (1998). Calibration of the Computer Science and Applications. Inc. accelerometer. Med Sci Sports Exerc.

[109] Sallis JF, Haskell WL, Wood PD, Fortmann SP, Rogers T, Blair SN (1985). Physical activity assessment methodology in the Five-City Project. Am J Epidemiol.

[110] Raynor DA, Phelan S, Hill JO, Wing RR (2006). Television viewing and long-term weight maintenance: results from the National Weight Control Registry. Obesity (Silver Spring).

[111] O’Neil PM, Currey HS, Hirsch AA, Malcolm RJ, Sexauer JD, Riddle FE (1979). Development and validation of the eating behavior inventory. J Behav Assess.

[112] O’Neil PM, Rieder S (2005). Utility and validity of the eating behavior inventory in clinical obesity research: a review of the literature. Obes Rev.

[113] Klem ML, Wing RR, McGuire MT, Seagle HM, Hill JO (1997). A descriptive study of individuals successful at long-term maintenance of substantial weight loss. Am J Clin Nutr.

[114] Raynor HA, Polley BA, Wing RR, Jeffery RW (2004). Is dietary fat intake related to liking or household availability of high- and low-fat foods?. Obes Res.

[115] Gorin AA, Wing RR, Fava JL, Jakicic JM, Jeffery R, West DS (2008). Weight loss treatment influences untreated spouses and the home environment: evidence of a ripple effect. Int J Obes (Lond).

[116] Jakicic JM, Wing RR, Butler BA, Jeffery RW (1997). The relationship between presence of exercise equipment in the home and physical activity level. Am J Health Prom.

[117] Cox JL, Holden JM, Sagovsky R (1987). Detection of postnatal depression. Development of the 10-item Edinburgh Postnatal Depression Scale. Br J Psychiatry.

[118] Lee KA, DeJoseph JF (1992). Sleep disturbances, vitality, and fatigue among a select group of employed childbearing women. Birth.

[119] Fairburn CG, Berglin SJ (1994). Assessment of eating disorders: interview or self-report questionnaire?. Int J Eat Disord.

[120] Evans C, Dolan B (1993). Body shape questionnaire: derivation of shortened “alternate forms”. Int J Eat Disord.

[121] Warren CS, Cepeda-Benito A, Gleaves DH, Moreno S, Rodriguez S, Fernandez MC (2008). English and Spanish versions of the Body Shape Questionnaire: measurement equivalence across ethnicity and clinical status. Int J Eat Disord.

[122] Kendall A, Olson CM, Frongillo E (2001). Evaluation of psychosocial measures for understanding weight-related behaviors in pregnant women. Ann Behav Med.

[123] Cohen S, Kamarck T, Mermelstein R (1983). A global measure of perceived stress. J Health Soc Behav.

[124] Walker LO (1997). Weight and weight-related distress after childbirth: relationships to stress, social support, and depressive symptoms. J Holist Nurs.

[125] Osborn CY, Weiss BD, Davis TC, Skripkauskas S, Rodrigue C, Bass PF (2007). Measuring adult literacy in health care: performance of the newest vital sign. Am J Health Behav.

[126] Cuellar I, Arnold B, Maldonado R (1995). Acculturation Rating Scale for Mexican Americans-II: a revision of the original ARSMA scale. Hispanic J Behav Sci.

[127] Yeh MC, Viladrich A, Bruning N, Roye C (2009). Determinants of Latina obesity in the United States: the role of selective acculturation. J Transcult Nurs.

[128] Wolfe WS, Sobal J, Olson CM, Frongillo EA (1997). Parity-associated body weight: modification by sociodemographic and behavioral factors. Obes Res.

[129] Harris HE, Ellison GT, Holliday M (1997). Is there an independent association between parity and maternal weight gain?. Ann Hum Biol.

[130] Phelan S, Smith K, Steele J-M, Wilt D, Ames S, McClure L (2010). What type of weight loss program do postpartum women want? Treatment preferences of postpartum women in two community settings. Calif J Health Prom.

[131] Smeeth L, Ng ES (2002). Intraclass correlation coefficients for cluster randomized trials in primary care: data from the MRC Trial of the Assessment and Management of Older People in the Community. Control Clin Trials.

[132] Raudenbush S, Bryk A (2002). Hierarchical linear models.

[133] Committee on Nutritional Status During Pregnancy and Lactation, Food and Nutrition Board, Institute of Medicine (1990). Nutrition during pregnancy.

[134] McKeown T, Record RG (1957). The influence of weight and height on weight changes associated with pregnancy in women. J Endocrinol.

[135] Wolfe WS, Sobal J, Olson CM, Frongillo EA, Williamson DF (1997). Parity-associated weight gain and its modification by sociodemographic and behavioral factors: a prospective analysis in US women. Int J Obes Relat Metab Disord.

[136] Gunderson EP, Quesenberry CP, Lewis CE, Tsai AL, Sternfeld B, Smith West D (2004). Development of overweight associated with childbearing depends on smoking habit: The Coronary Artery Risk Development in Young Adults (CARDIA) Study. Obes Res.

[137] Gunderson EP, Murtaugh MA, Lewis CE, Quesenberry CP, West DS, Sidney S (2004). Excess gains in weight and waist circumference associated with childbearing: The Coronary Artery Risk Development in Young Adults Study (CARDIA). Int J Obes Relat Metab Disord.

[138] Gunderson EP, Rifas-Shiman SL, Oken E, Rich-Edwards JW, Kleinman KP, Taveras EM (2008). Association of fewer hours of sleep at 6 months postpartum with substantial weight retention at 1 year postpartum. Am J Epidemiol.

[139] Howard BV, Van Horn L, Hsia J, Manson JE, Stefancik ML, Wassertheil-Smoller S (2006). Low-fat dietary pattern and risk of cardiovascular disease: the Women’s Health Initiative Randomized Controlled Dietary Modification Trial. JAMA.

[140] Pituch KA, Stapleton LM, Kang JY (2006). A comparison of single sample and bootstrap methods to assess mediation in cluster randomized trials. Multivar Behav Res.

[141] Olson CM, Strawderman MS, Hinton PS, Pearson TA (2003). Gestational weight gain and postpartum behaviors associated with weight change from early pregnancy to 1 y postpartum. Int J Obes Relat Metab Disord.

[142] Kumanyika SK, Obarzanek E (2003). Pathways to obesity prevention: report of a National Institutes of Health workshop. Obes Res.

[143] Kac G, Benicio MH, Velasquez-Melendez G, Valente JG, Struchiner CJ (2004). Gestational weight gain and prepregnancy weight influence postpartum weight retention in a cohort of brazilian women. J Nutr.

[144] Abrams B, Altman SL, Pickett KE (2000). Pregnancy weight gain: still controversial. Am J Clin Nutr.

[145] Kuhlmann AK, Dietz PM, Galavotti C, England LJ (2008). Weight-management interventions for pregnant or postpartum women. Am J Prev Med.

[146] Walker LO, Kim S, Sterling BS, Latimer L (2010). Developing health promotion interventions: a multisource method applied to weight loss among low-income postpartum women. Public Health Nurs.

[147] National Task Force on the Prevention and Treatment of Obesity (2000). Overweight, obesity, and health risk. Arch Intern Med.

[148] Institute of Medicine (US) and National Research Council (US) Committee to Reexamine IOM Pregnancy Weight Guidelines (2009). Weight gain during pregnancy: reexamining the guidelines. Rasmussen KM, Yaktine AL, editors.

[149] Phelan S (2010). Pregnancy: A “teachable moment” for weight control and obesity prevention. Am J Obstet Gynecol.

[150] McBride CM, Ostroff JS (2003). Teachable moments for promoting smoking cessation: the context of cancer care and survivorship. Cancer Control.

